# Royal Infirmary of Edinburgh

**Published:** 1829-07-01

**Authors:** 


### CLINICAL REVIEW.

### XXXVII.

**ROYAL INFIRM. OF EDINBURGH.***

Simple and Compound Fractures.

It gives us much pleasure to meet Dr. Ballingall again on the tapis, to learn the results of his experience, and peruse his instructive and interesting observations. We believe we were the first to discover the merit of these lectures, and we gave them a publicity and extent of circulation which they never could attain in their native form. At pre-

* Clinical Lecture delivered by Dr. Ballingall, Feb. 26, 1829.

sent, we are gratified to find that our contemporaries have also appreciated their value, and transferred them, in no niggard fashion, to their pages. This is as it should be, and we wish, in all sincerity, that more provincial surgeons than our friend Dr. Ballingall would strive to earn the same honorable meed of approbation. It was said, we believe by Mr. Brougham, that the rising generation wear their spurs at their toes, and woe to the posterior parts of those who are lagging before them. Would to Heaven that the spur was applied now and then to our country surgeons, who ought to be rather in the van than the rear of the present intellectual march. But to our analysis; and first we shall notice the subject of fractures, which, indeed, forms the commencement of the present lecture. Dr. Ballingall shall speak for himself.

"Of these acute cases, the fractures of the limbs have, upon the present, as upon former occasions, constituted a large majority; of simple fractures I find that there have been treated, on my side of the House, twenty-six cases, all of which have either terminated successfully, or are in the progress of cure, with the exception of that of *William Fernie*, æt. 62, who was admitted on the 3d of January with a fracture of the neck of the femur. This man was brought from Kinglassie in Fifeshire, where he had met with the accident about ten days before. His limb was immediately placed in Boyer's splint, and was extended to the same length as its fellow of the opposite side; but although the old man complained of no inconvenience from the apparatus, it was soon perceived that ulceration had taken place on the sacrum to a considerable extent. This induced me to remove the splint and to place the patient on his side; here again a gangrenous spot appeared over the trochanter of the right thigh, and the patient was subsequently turned to the opposite side, my object now being to obviate the effects of pressure, and the extension of the gangrene, rather than to look for any successful treatment of the fracture. The ulceration, however, continued to extend, and the patient's strength to sink, notwithstanding the liberal exhibition of animal food, porter, and wine, until the 4th of February, when he died, six weeks after the receipt of the injury; and, I regret to say that the body was immediately removed to Fife without affording us an opportunity of examining the state of the broken bone.

"In the cursory observations which I have had occasion to lay before you on the subject of fractures, I have always inculcated the necessity of giving your attention to the general principles on which fractures are treated, observing that the variety of these accidents is so endless as to render it a matter of impossibility to be previously acquainted with the specialities of each individual case. My views of the treatment of fractures of the neck of the femur, I explained to you from one of my printed Lectures, and I would now only observe, that the result of the foregoing, as well as of some other cases of the same kind which I have witnessed, has made me lately disposed to question whether we would not do well, in many cases of fracture of the neck of the thigh-bone to be satisfied with the simple treatment recommended by Sir A. Cooper; to place the limb in a relaxed position with a folded pillow under the knee-joint, and to take our chance of such a cure as nature may be pleased to afford us, rather than by confining the patient in a coercive apparatus to run the risk of inducing ulceration or gangrene, the progress of which, in advanced life, or in broken constitutions, it is so little within our power to control." 4.

Three cases only of compound fracture occurred during the session, and of these but one deserves notice here.

*Case.* Alexander Kerr, ætatis 20, was overturned in a cart on the morning of the 6th of October, when the edge of the vehicle, the whole weight of the horse resting on the shaft, fell against the lower part of his leg. At the time of his admission into the Royal Infirmary the whole left leg was tense, much swelled, and painful. About an inch and a half above the ankle was a small wound over the anterior surface of the tibia, and the skin above the outer malleolus was very much contused and fluctuated from the effusion of blood beneath it. When the foot was moved an indistinct crepitus was felt. We cannot say that the Doctor's description is particularly lucid, for no one on reading it can venture to pronounce either where or what the fracture was. However the limb was placed upon "M'Intyre's splint," and the spirit lotion applied. At night he took an anodyne, castor oil next morning, and an antimonial solution was administered internally from time to time. On the 8th we find the following report.

"Slept ill; pulse 108, very full and strong; bowels once relieved; tongue slightly furred; skin upon the outer side of the leg is evidently gangrenous, and that upon the inner side considerably discoloured. An incision was made through the former, some serum was discharged, but the wound bled very little. He was bled to twenty ounces." At 11, p.m. the pulse was 132; the "gangrene" did not appear to have spread upon the outside, but the skin on the inside was more extensively discoloured. He was bled again to ℥x. after which he slept a little, and on the 9th was improved. The pulse was 112, and not so strong; the skin was hot; the thirst less; the bowels once relieved. The "gangrene" had not spread at all upon the outside of the limb, and there was some healthy purulent discharge from the incision; on the inside the gangrene had extended a little. The last blood drawn was neither buffed nor cupped. The fermenting poultice was applied to the leg and the antimonial solution continued. The feverish symptoms now subsided, and on the 11th the pulse was 100; skin cool; tongue less furred; appetite returning; "the gangrene had ceased to spread, but the sloughs had not begun to separate."

"It now became obvious that we should not be compelled to amputate by the extension of the gangrene, but when the sloughs came to be detached and both the tibia and fibula were laid bare to a considerable extent, with a copious purulent discharge, and rather profuse sweats, I was greatly inclined to amputate the leg; and had not some of my colleagues entertained a more favourable opinion of the case than I did, it is probable the operation would have been proposed to him.

"There was great encouragement however to persevere in our attempts at a cure, from the prosperous state of the man's general health; as well as from his youth, and apparently vigorous habit. The extensive sore which nearly surrounded the lower part of the limb became covered with florid and healthy granulations; the discharge rapidly diminished, and on the 21st December he was discharged cured, a trifling exfoliation having previously taken place from the fore part of the tibia."

We have often been surprised at observing the very loose way in which surgeons employ the terms mortification and gangrene. If a leg after an accident begins to be blackish and discoloured, and especially if vesications filled with dark modena-coloured serum form upon it, the case is set down instanter as a case of gangrene. Now the fact is, that a very large proportion of this kind of cases are neither gangrene nor likely, under proper treatment, to end in it, and therefore it is clearly a dangerous error to consider them as such. A man receives a simple or a compound fracture, perhaps of no great severity—the limb is put up in the apparatus determined on, whatever it may be—and for one or two days would seem to be doing tolerably well. About this time, however, a good deal of pain is experienced in the limb, and on opening the apparatus, the leg (for we will suppose the tibia to have been broken in its shaft) is discoloured in patches of brown and yellow, from the ankle to the knee. In one or more parts, generally on the fore part of the leg, the cuticle is raised in vesications, filled with serum of port wine colour, and on breaking these the cutis itself looks mahogany-coloured, or perhaps of its natural tint below. The patient all this time may be little disturbed in his general health, or instead of the prostration consequent on mortification, may have a full bounding pulse, a hot flushed skin, a loaded tongue, in fact, the genuine symptoms of excitement.

Such a case as this may appear to a person who has not seen many of the same description, as one that is fraught with imminent peril, in fact, as a case of traumatic gangrene. But indeed it is not of so dangerous a character, and under attentive and soothing local treatment, with free depletive measures, if the case has been one of the sthenic character, the skin which has perished will come away, the leg will recover itself, and all will do well. We cannot help thinking that the case recorded by Dr. Ballingall as one of traumatic gangrene, approached much nearer to that which we have described, than to what he has considered and termed it. We see, for our own parts, no evidence of gangrene in that particular instance; the limb was not cold; it did not die; the sloughing was superficial; the pulse was not weak, rapid, or intermitting, but full and strong; the recovery from the gangrenous state was speedy, and that under *bleeding* and depletory measures; in short, it was not a case of mortification requiring amputation, but an instance of inflammation set up in the skin, and perhaps the cellular membrane at the seat of injury, ending in superficial sloughing, but implicating generally neither the limb nor the constitution. In the following remarks of Dr. Ballingall, we, for the most part, perfectly agree, and we are sure that those who have seen most of these bad accidents, will deprecate with him the exclusive and narrow views which some take of their character and treatment.

"Of compound fractures I have hitherto said but little in these lectures, nor do I now propose to enlarge, for although in the two cases just mentioned you have witnessed a successful termination under the treatment adopted, yet they are, upon the whole, a class of accidents the results of which have been to me the least satisfactory of all that I have had occasion to treat in this house, and are not co-incident with my experience of them in other situations.

"Had these results been the consequence of one uniform mode of treatment, I should naturally have concluded that that treatment was erroneous. But when I look back to the mode of dressing the wounds—sometimes by a piece of lint soaked in blood, and allowed to form an encrustation over them—sometimes by covering them with a paste of gum, or a pledgit of simple dressing, and sometimes by bringing their lips into accurate apposition with adhesive straps:—When I look again to the different positions in which the limb has been placed—either closely enveloped in splints—lying less constrained in a fracture box, or simply resting on a pillow—sometimes in the bent—sometimes in the extended position:—And when I look also to the various means taken to subdue the violent inflammation and high symptomatic fever accompanying these injuries; by general, or by local bleeding, according to circumstances; very frequently by the use of cold evaporating lotions to the seat of the injury, and sometimes by the use of anodyne fomentations or cataplasms; I cannot admit that the unfavourable results which I have so often had occasion to deplore have been attributable to any bigoted prejudices or exclusive partialities in the mode of treatment.

"In my remarks upon this subject, I took occasion to observe that we are greatly in want of a work on these accidents, from some surgeon of varied and extensive experience. I say varied experience, because it appears to me that we are often led by the irresistible force of habit to give our attention too exclusively to one mode of treatment. I remember to have heard an hospital surgeon assert that he never expected to lose another case of compound fracture, by following up the practice, which seemed to him to be a new one, of closely enveloping such fractures in splints and bandages, without undoing them for weeks together. But it is not from those who take such a limited or exclusive view of this matter that we are to expect such a work as I could wish to see, but from those who are capable of discriminating between those cases (perhaps numerous ones) in which the above practice is advantageous, and those in which it is not only injurious, but absolutely insufferable.

"Before quitting this subject I would beg leave to mention that there are two points in the treatment of compound fractures upon which my own observation has led me to form a very decided opinion, and to offer you a remark which may possibly prove useful hereafter. I have, I think, too frequently seen a reluctance to use the saw in removing the protruding extremities of the bone, when these were either difficult to reduce, or of a sharp and spicular form; and I have, I think, sometimes seen the closure of the external wound attempted by means too forcible and too long continued."

We do indeed require a monograph upon the subject of compound fractures, and not the catch-penny production of a puffing adventurer, but one that shall be the result of extensive and varied observation. It is only the surgeon of a great hospital who could give us such a work as is wanted. We must now take our leave of the able lecturer for the present, but much remains behind that is highly deserving of attentive consideration. To this we shall return as opportunities present themselves, and again we beg leave to part from Dr. Ballingall with feelings of sincere respect.

---

### XXXVIII.

### LVIII.

### EDINBURGH ROYAL INFIRMARY.*

### 1. PRIMARY AMPUTATIONS.

*Case* 1. Robert M'Gregor, fifteen years of age, was admitted late in the evening of the 28th of January, having had his right arm drawn into the machinery of a paper-mill at 5 P. M. The arm was torn off from the body about the middle of the humerus, and the extremity of the bone projected from the muscles. The laceration extended on the inside into the axilla, and destroyed the inferior margin of the pectoralis major and latissimus dorsi. The skin for a short way on the back of the scapula was destroyed, together with a considerable part of the clavicular portion of the deltoid. The artery was seen pulsating about three inches from its end, which was closed by coagulated blood. No hæmorrhage had taken place, but the extremities were cold and the pulse feeble.

Amputation at the shoulder-joint by making a single flap of the deltoid was performed by Dr. Campbell, and four vessels only required to be secured. An opiate was administered and the patient went on well till the night of the 1st of February, when much secondary hæmorrhage took place from a small vessel which was therefore tied. At half past 2 in the morning the ligature came away from the axillary artery, and bleeding followed to a material amount before the vessel was secured. An anodyne was given, and the pulse was reported at 130, but stronger than on the preceding evening. For several days the patient was considered in imminent hazard, some sloughing having taken place from the edges of the wound, with a copious discharge of ill-conditioned matter, and the pulse ranging from 125 to 150, with occasional rigors, and profuse sweats. The local treatment consisted in daily dressing with resinous ointment; the general, in the free exhibition of opiates, beef tea, and small quantities of wine. The cure went on without interruption or untoward event, the lad's appearance amended greatly, and Dr. Ballingall, at the period of his lecture, considered him as out of the wood.

The following remarks are so just, that we cannot refrain from transcribing them without abbreviation.

"In my comments upon this case, I viewed it as illustrative of three important points, the spontaneous cessation of the hæmorrhage, in cases where limbs have been torn from the trunk; the question of primary and secondary amputation; and lastly, the mode of performing this operation at the shoulder-joint.

* *Clinical Lecture by Dr. Ballingall.*

"In illustration of the first point, I showed you the portion of the humeral artery which was removed along with the remains of the arm, the internal coat of which was in some points slightly lacerated, while the external coat was over-stretched, and a considerable portion of it filled with coagulum. I referred you for farther illustration of the state of arteries after accidents of this kind, to a case detailed in the 19th volume of the Edinburgh Medical Journal, by Mr. Lizars, and to a very valuable paper, containing indeed all the information which we possess on this interesting subject, by Professor Turner, in the Medico-Chirurgical Transactions of this place. By the kindness of this last mentioned gentleman, I was also enabled to show you some drawings, exhibiting the state of arteries in accidents of this nature; where, in some cases, we find their internal coats completely torn through, and corrugated, or coiled up, as it were, within the vessel, so as to prevent the effusion of blood.

"On the subject of primary and secondary amputation, we now possess a series of valuable observations, from the time at which it was made the subject of a prize question by the French academy in 1756, down to the present day: when the advantages of primary amputation are, I believe, admitted by every practical surgeon, who is now enabled to add to his own experience that of the French army surgeons, as detailed in Baron Larrey's writings, that of the English army surgeons during the peninsular war, as detailed by Mr. Guthrie and Dr. Hennen, and that of the naval surgeons, as detailed in Mr. C. Hutchison's work. One of the most striking illustrations of the successful issue of primary amputations, is contained in an extract from a report made by Dr. Burke, inspector of hospitals to his Majesty's forces in Bengal, which has been made public by my friend Mr. Annesley, in his splendid work on the diseases of India. Dr. Burke states, that 'of eighty cases of amputation,' performed at Bhurtpore in Upper India, 'the whole recovered in fourteen days.'

"You must not, however, Gentlemen, expect to meet with the same proportional success in the amputations performed in civil hospitals; at least, I am entitled to say, that the contrast between the success of primary and secondary amputations during the time that I have served in this house, has by no means resembled that which I was accustomed to see and to hear of in the army. For these different results in military and in civil hospitals, many reasons might, I think, be assigned, some of them more satisfactory than those mentioned by Sanson, who, in a recent paper upon this subject, has noticed the fact, but has, I think, failed to give a very luminous or satisfactory explanation of it.

"In speaking of the operation at the shoulder joint, I remarked, that although this operation has undergone many modifications, to some of which the names of Morand, La Faye, Le Dran, Larrey, Lisfranc, Broomfield, Alanson and others, have been attached, yet these may all be resolved into two modes of proceeding, either by forming a superior and inferior, or an anterior and posterior flap. I have myself operated in both ways, but not sufficiently often to enable me to institute any fair comparison as to the best mode of operating; I may however be permitted to remark, that in a very large proportion of the cases requiring amputation at the shoulder joint, the soft parts are so lacerated as to leave us no choice, but to compel us, as in the present case, to form the flaps as circumstances best will admit. We are now well aware that the apprehension of an uncontrollable hæmorrhage, which alarmed our predecessors and made them slow to adopt this operation, is altogether unfounded; the bleeding may always be controlled by firm pressure above the clavicle, by the hands of a steady assistant; and, in fact, this compression, as my distinguished predecessor Dr. Thomson observes, has been found 'easier in practice than it appeared to be in speculation.' It is necessary, however, to remark, that at the moment of cutting through the axillary vessels and nerves, the patient is apt to give an involuntary start, and may throw the fingers of the assistant off the artery; an accident which once happened to a gentleman assisting me in this operation, and by which I nearly lost my patient. I was for a moment completely blinded by the discharge of blood into my eyes from the open axillary artery."

*Case* 2. Charles Cuthbertson, ætatis 71, was admitted on the 18th of December with a comminuted fracture of the radius and ulna at their carpal extremities, an extensive lacerated wound of the left wrist, and a severe contusion in the lower part of the back, with a fracture of some of the ribs.

"Although, as I stated to you in the theatre at the time of the operation, I entertained little or no expectation of this man's recovery, I determined, after a few moments consultation with my colleagues, to remove the arm a little below the elbow. It was obvious, however, at the first dressing, that no prospect of union existed; the lips of the wound, instead of presenting that wholesome turgidity and tension which presents itself in a healthy stump, were flaccid, somewhat livid, and apparently tending to gangrene; the smaller vessels (if I may use the expression) had not taken up, but had poured out a quantity of grumous blood which flowed through the dressings; at the same time the patient began to complain of oppressed breathing, and symptoms of inflammatory action within the thorax, so that he had to contend with all those difficulties which you may suppose to exist in the case of an old man labouring under acute inflammation within the trunk, and gangrene in one of the extremities. Amidst a choice of evils, the abstraction of a limited quantity of blood was tried but without any good effect, and the patient died during my temporary absence in London, about eight days after the receipt of the injury. No report of the dissection appears to have been entered in the register, but I am told that the whole of the ribs on the right side, with the exception of the two inferior ones, were found fractured, and some recent deposition of lymph on the pleuræ.

"This case, Gentlemen, I am induced to mention, not from any very instructive lesson to be gathered from its progress, much less for any thing very unexpected in its event, but to caution you against an accident which occurred during the operation. While passing the knife through the fore-arm, from the radial to the ulnar side, for the purpose of forming a posterior flap from the extensor and supinator muscles, its point slipped between the bones, which rendered it necessary to withdraw it and to pass it more carefully behind the ulna; this was perhaps owing partly to my own inadvertence to the exact position of the bones, but partly, I believe also, to the connection between the lower extremities of the radius and ulna having been broken up, so that in pressing the knife close to the back of the radius, with the view of obtaining a sufficiency of muscular substance to form a good flap, the parallelism of the two bones was in some measure destroyed, the radius was pressed forwards, and the interosseous space thus presented to the point of the knife."

### II. SECONDARY AMPUTATION.

*Case* 1. Helen Coghill, æt. 21, was admitted on the 8th of October, with the left knee swelled, hot and painful; severe pain in the condyles of the femur on moving or pressing the patella; limb retained always extended; pulse natural; tongue clean; bowels regular. She stated that after a fall, received ten years previously, the joint was first inflamed, and eighteen months ago, after a similar accident, the inflammation was much increased, and had been more severe than even in the seven weeks preceding her admission. Leeches, opium, moxas, &c. were the means employed, but without the least effect in arresting the disease. The sufferings of the patient were very severe, the constitution sympathised, and under these circumstances that dernier resort, amputation, was peformed on the 5th of December. The joint, on examination, was found to be extensively diseased, its cartilages being either completely destroyed, or detached altogether from the bones. The patient for some days was harrassed with nausea and occasional retching, with her pulse varying from 136 to 140. The general appearance, notwithstanding, was improved, and although superficial ulcerations took place upon the back, the stump put on a very promising appearance, and the greater part united by the first intention. On the 3d of January she left the house, but after this, small phlegmonous swellings formed and burst in the line of the cicatrix.

"You would observe, Gentlemen, that in amputating this young woman's thigh, instead of forming two lateral flaps, as I had hitherto been accustomed to do, I formed an anterior and posterior flap, the one from the extensors on the fore part, and the other from the flexors on the back part of the thigh. This is a mode of operating which I first saw practised by Mr. Liston in the cases of two boys whom he operated upon in the house last autumn; and in the case of such young subjects who cannot readily be made to retain their stumps in a desirable position, but are constantly inclined to elevate the point of the stump, it appears to me to offer decided advantages; in the first place, it obviates the projection of the bone between the lateral flaps, which I am told has sometimes occurred, and, in the next place, you will see that the more the point of the stump is elevated, the more are the extensor muscles relaxed so as to afford a covering for the point of the bone. Other collateral advantages attendant upon this mode of forming the flaps are pointed out in a paper of Mr. Creasers, formerly of the Bath Infirmary, in the 22d volume of the Edinburgh Medical and Surgical Journal, although he indeed recommends the flap to be formed by cutting from the surface towards the bone, instead of transfixing the limb and cutting outwards.

"The plan of dressing stumps after the common circular amputation of the thigh, so as to place the line of the cicatrix transversely instead of perpendicularly, you will find advocated both by Mr. Guthrie and by Mr. Copland Hutchison, the latter of whom, in the first edition of his Surgical work, gave a marginal sketch well calculated to illustrate his valuable remarks upon this point."

*Case* 2. J. Browne, æt. 37, a seaman, having lost all his toes in consequence of exposure to great cold twelve months previously, on the coast of Norway, was admitted with extensive ulcer and destruction of skin on the under surface of the left foot. Dr. Ballingall being satisfied that no firm or permanent cicatrix was likely to be be formed unless the metatarsal bones were removed, performed an operation for that purpose on the 4th of November. It consisted in making an incision convex anteriorly from a little beyond the base of the first metatarsal bone to the most extreme point of the base of the fifth, and forming a flap from the integuments on the dorsum of the foot. The four outer metatarsal bones were then disarticulated, the internal cuneiform sawn through, and a corresponding flap formed from the integuments of the sole. The two flaps thus obtained were united by three points of the interrupted suture. Partial union by the first intention was procured, but an attack of the erratic erysipelas succeeded, which protracted the cure. However, he was able to leave the infirmary on the 5th of January, for London. Had the great destruction of the soft parts in the sole not proved a bar to such a proceeding, Dr. B. would have greatly preferred a large single flap from that part turned up over the anterior extremities of the tarsal bones, to the double one, which circumstances forced him to adopt. The cicatrix on the dorsum of the foot is much the least liable to injury in walking.

### III. Recto-Urethral Fistula.

Every one must know how troublesome a class of cases are these fistulous communications between the pelvic outlets of the body. The recto-vesical, rectovaginal, and recto-urethral fistulæ are too often the opprobrium chirurgicum, and productive of more misery in the minds of patients than what might be deemed more serious maladies. The particulars of a case reltated by Dr. Ballingall are of practical importance, as shewing, what too rarely happens, the cure of the disease by the actual cautery.

Walter Hay, æt. 45, who was cut in the hospital for fistula in ano, and discharged almost well, returned with a difficulty in passing his water, the half of which was discharged from the anus. A large catheter was easily passed into the bladder and the right lobe of the prostate found to be enlarged, hardened, and painful upon pressure. The introduction of the speculum ani distinctly shewed the orifice of a sinus between the urethra and rectum, and a probe directed through it was made to grate upon a catheter previously passed into the bladder. On the 10th of November, the patient was taken into the operating theatre, and a full sized catheter attempted to be introduced, but without success, in consequence of the inflammation and swelling excited by the former examinations. The rectum, however, being well dilated with the speculum, the callous edges of the fistula were cauterized by the red hot iron. Next day fourteen leeches were required on account of pain about the parts, but on the night of the 12th, the swelling, &c. had so much abated that an elastic catheter was secured in the bladder, in which it was continued for upwards of a fortnight. The whole of the urine was evacuated through the instrument, but the latter producing a puriform discharge from the urethra, was withdrawn at the end of the before-mentioned time. The discharge in question gradually subsided, and on the 6th of December the patient was dismissed cured.

"The cure," says Dr. Ballingall, "from a single application of the cautery was more than I anticipated, and was no doubt greatly facilitated by the free and pervious state of the urethra, and by our being enabled to introduce a full-sized catheter into the bladder soon after the application of the cauterizing iron, before the eschar occasioned by it was detached. But while this open state of the natural passage obviously contributed much to the cure, it presents to me a very considerable difficulty in accounting for the formation of this fistulous opening, which was more than an inch within the extremity of the gut."

However difficult the rationale of the case may be, it is certainly true that abscesses connected with the urethra, fistulæ, and even formidable extravasation of urine will sometimes occur when no stricture can be found in the canal. They depend probably on the same causes which occasion inflammation and its consequences elsewhere, although the single and preponderating one of stricture is apt, and naturally so, to attract the whole of our attention. Abscesses form in the neighbourhood of the rectum, and open into its cavity without any mechanical obstruction of the gut, and we see no good reason why the same should not sometimes be the case with the urethra. Its rarity is to us more surprising than its occurrence.

